# How can we make GPS tracking studies more open, reproducible, and collaborative? A vision for the OpenGPS platform

**DOI:** 10.1016/j.dib.2025.111603

**Published:** 2025-05-02

**Authors:** Milad Malekzadeh, Hui Jeong Ha, Katarzyna Sila-Nowicka, Vanessa Brum-Bastos, Jinhyung Lee, Urška Demšar, Jed A. Long

**Affiliations:** a Digital Geography Lab, Department of Geosciences and Geography, University of Helsinki, Finland.; b Department of Geography and Environment, Western University, London, Ontario, Canada; c School of Environment, The University of Auckland, Auckland, New Zealand; d School of Earth and Environment, University of Canterbury, Christchurch, New Zealand; e School of Geography and Sustainable Development, University of St Andrews, St Andrews, Scotland, United Kingdom

**Keywords:** Tracking, Trajectory, Movement, Open data, Spatial-temporal, GPS, Human mobility

## Abstract

This paper introduces OpenGPS, a platform envisioned for the archiving and processing of human mobility GPS data. The OpenGPS addresses the need for a centralized, privacy-preserving system that securely stores, shares, and analyzes GPS tracking datasets. The platform is envisioned to develop in three phases. Phase I focuses on collecting metadata from existing GPS tracking studies worldwide, providing a foundation for future research. Phase II involves archiving GPS data with standardized formats and robust privacy safeguards, ensuring data is accessible while protecting individual privacy. Phase III integrates advanced analytical tools and workflows directly into the platform, enabling efficient analysis and fostering collaboration among researchers. The OpenGPS aims to overcome the limitations of current human mobility studies by offering a standardized repository that enhances reproducibility and openness in research. By facilitating the sharing of data and methodologies, the OpenGPS will promote new insights and innovations in human mobility research. This platform is poised to become a critical resource for the scientific community, bridging gaps in data availability, and enabling comprehensive meta-analyses across different geographical and temporal scales. Through OpenGPS, researchers can collaborate more effectively, share resources, and advance the understanding of human mobility patterns globally.

## Highlights

• OpenGPS addresses challenges in data sharing, reproducibility, and collaboration.
• A three-phase plan: metadata collection, data archiving, analysis tools.
• It ensures privacy with multi-tiered access, encryption, and anonymization methods.
• Standardized formats and governance framework support open and FAIR data practices.
• It fosters global research, enabling large-scale meta-analyses in mobility studies.

## 1 Introduction

On May 1, 2000, the United States government turned off the intentional degradation of GPS signals, a process known as selective availability, which was used to limit the accuracy of GPS for civilian use. Following this change, researchers began to leverage modern GPS technology [1] to study human mobility and transportation patterns in time across space. GPS technology, often used interchangeably with GNSS (Global Navigation Satellite Systems), refers to the global network of satellites used for positioning and navigation, including systems such as GPS itself (Global Positioning System, USA), Galileo (European), Beidou (Chinese), and Glonass (Russian). It is the de-facto standard for collecting high spatio-temporal resolution data on human mobility; either using standalone devices [2] or GPS receivers integrated into mobile phones and other smart devices [3,4]. GPS tracking data, also referred to as GPS movement data, has provided notable improvements over traditional travel surveys by reducing under-reporting of trips [5], minimizing recall bias [6] and improving spatial-temporal precision [1], while at the same time using automated data collection to decrease the time-burden of surveying participants.

In recent years, telecom and tech companies have developed proxy datasets used to study human mobility (e.g., Google Mobility reports, Apple Mobility trends). Additionally, various mobile applications, including those for weather forecasting, navigation, fitness tracking, and social networking, passively collect high-resolution GPS data from users [[7], [8], [9]], often without explicit mobility-focused intent. These proxy datasets aggregate individual level data collected by smart devices to represent population trends within specific administrative or more arbitrary boundaries. The widespread use of these datasets became evident in studies on the impact of human mobility (and changes therein) on the spread of COVID-19 [10,11] and inequities (i.e., the luxury of social-distancing) during the pandemic [12,13]. Despite their large sample sizes, which can sometimes represent millions of individuals, these proxy mobility datasets lack the individual-specific and contextually relevant patterns necessary to address fundamental questions about human mobility in social science. The absence of individual-level socio-demographic information and the aggregation to larger geographic units contributes to the shortcoming in addressing such questions [14,15].

Smaller scale (i.e., < 5000 participants) studies of human mobility using high spatio-temporal resolution GPS data continue to provide new insights about how individual movement is related to various societal concerns and urban processes. Unlike the large proxy datasets that have become more prominent during the pandemic, these smaller scale GPS-based studies have a much longer history [16]. Such targeted GPS tracking studies, as commonly employed by researchers, have the significant advantage of simultaneously collecting individual-level information on mobility patterns [17], urban context [18], health [19], and well-being [20]. Modern GPS tracking studies often combine fine-scale location data with real-time questionnaires that can be triggered by location (e.g., proximity to a restaurant) – termed geographical ecological momentary assessments [21]. This combination allows researchers to address much more targeted and fundamental questions related to mobility indicators and patterns, while simultaneously controlling for other known effects and confounding factors. However, the GPS data collection process can be intrusive, time-consuming, and expensive, making it impractical for many researchers, particularly those with limited resources. Consequently, researchers facing such constraints have two viable alternatives: they can either utilize existing public datasets, or they can engage in partnerships with colleagues who already possess the requisite GPS data.

Given the rich information provided by GPS data on individuals, safeguarding geoprivacy during data collection requires strict ethical considerations with respect to sensitive individual location information (and the associated individual-level socio-demographic variables). For this reason, almost no GPS tracking datasets are shared publicly, but some can be accessed through the researcher (e.g., [22]). This lack of publicly available data has severely limited open and reproducible research practices in studies employing GPS tracking data, despite this being an emerging and important area of geographical analysis [23]. While researchers are generally willing to share their data when appropriately credited, several challenges persist [24]. Many GPS tracking datasets remain confined to local storage, often accessible only to the primary investigator, raising the risk of irreversible data loss over time. Further, this lack of data sharing and openness has hindered more collaborative work on human mobility data. Notably, there is a clear lack of development of more substantive meta-analyses of human mobility patterns combining multiple studies, across spatial and temporal scales, in different geographical contexts. There is a clear opportunity to develop an archive of past, present, and future GPS tracking studies to foster collaboration between researchers, facilitate data sharing and standards, and provide a system for archiving these rich datasets in an effort to create a more reproducible and open model for the science of human mobility.

In this paper we provide a vision for the first platform for archiving first metadata, and then GPS data, from GPS tracking studies which we call the OpenGPS. The objective of the OpenGPS is to develop a secure, privacy preserving system to archive, share, and analyze GPS tracking datasets for the study of human mobility patterns. These datasets typically include high-resolution spatiotemporal trajectories (i.e., sequences of timestamped geographic coordinates) and are often accompanied by additional contextual information, such as participant demographics, environmental conditions, or sensor data (e.g., accelerometer, speed). While generic repositories like Zenodo or Figshare exist, they lack specialized features for the high-resolution, privacy-sensitive nature of GPS human mobility data and do not provide specialized tools such as in-platform trajectory analysis, anonymization protocols tailored for high-resolution movement data, or integrated context-aware analysis pipelines. The development of OpenGPS will enable new opportunities for collaborative work in the study of human mobility and fast-track new insights and analytical methods for these rich datasets, much in the same way the development of the Movebank Database (Box 1) fast forwarded developments in wildlife movement ecology by centralizing the storage and sharing of wildlife tracking data [25].

Box 1 **The MoveBank Database**

**Fig. B.1:**
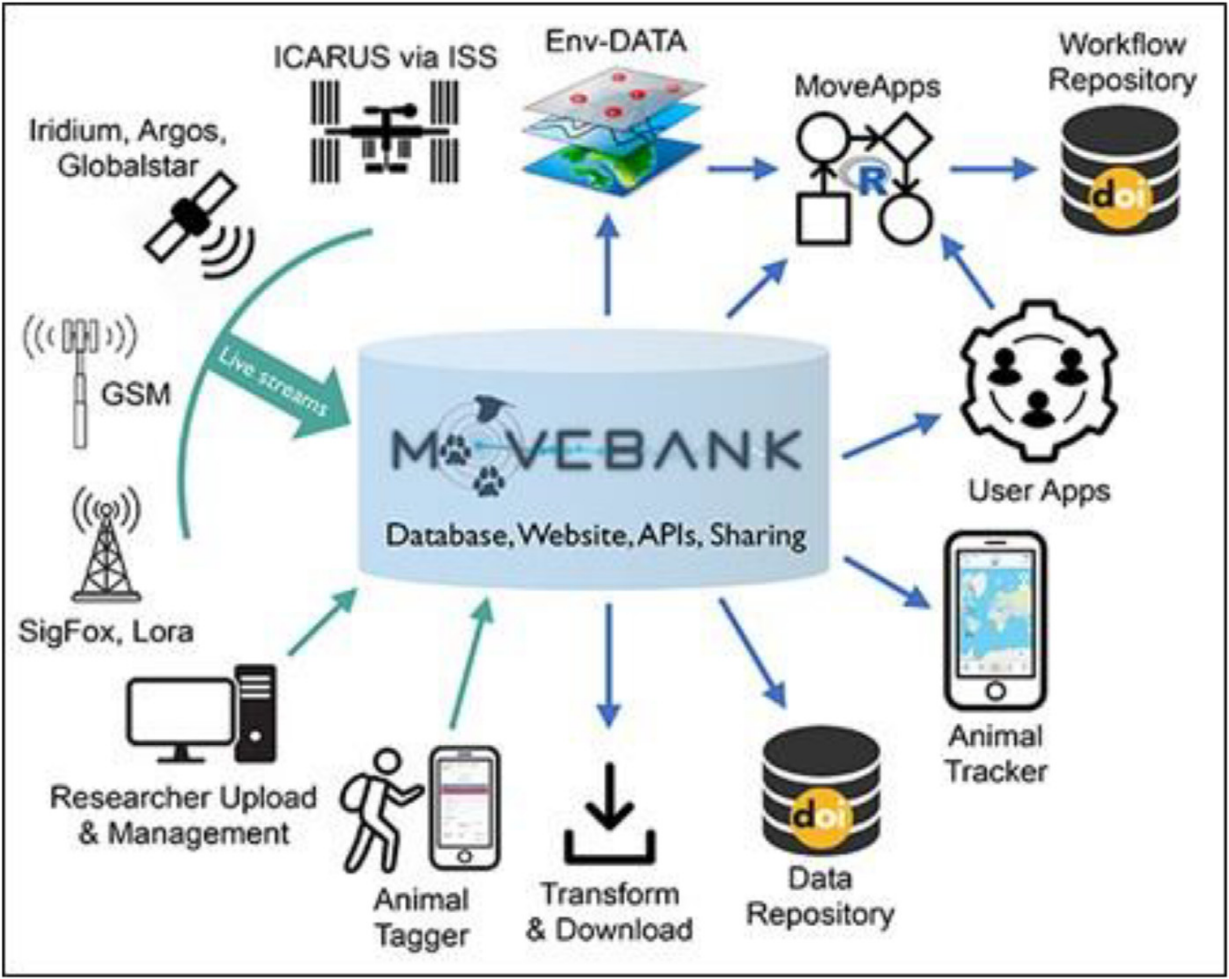
Schematic overview of the Movebank cyberinfrastructure ecosystem, illustrating the integration of various tracking technologies and data sources into the central Movebank database. For details of the Movebank components, refer to https://www.movebank.org/cms/movebank-content/why-use-movebank. The figure is adapted from [25].

Movebank is a platform for storing and sharing animal tracking data, which supports data archiving in a standardized format, offering various levels of data openness and facilitating large-scale, collaborative analyses [25]. Since its introduction in 2007 it has grown to host data from over 8000 studies, capturing the movement of more than 1000 species, contributed by over 4000 data owners. At present, Movebank contains over 6.2 billion animal location data points. The platform supports collaborations among researchers using data from different species and geographies, leading to significant advances in animal movement ecology, such as for example a global study of terrestrial mammal movement [50] or responses in animal movement to climate and environmental changes across the Arctic [51]. Datasets are stored in a standardized format [52], are available globally, and can be viewed instantaneously on a map interface within the Movebank platform: https://movebank.org. At the same time, the system allows data owners to assign various levels of accessibility, from fully open (and downloadable), to available upon request, to fully private. No equivalent to Movebank exists for human mobility GPS studies, and to our knowledge, nothing like this has ever been attempted. (Fig. B.1)

Alt-text: Unlabelled box

## 2 The OpenGPS Architecture

The development of a centralized archiving and processing platform for GPS tracking studies will benefit the human mobility community in two different ways. First, currently there is no centralized archiving platform for studies that have collected GPS tracking data to study human mobility. We envision the OpenGPS as a hub for human mobility GPS studies, storing fundamental information such as where and when studies were undertaken, and other details about study design. This repository will serve as a new resource for designing future GPS studies, and for identifying key gaps in the current distribution of data and existing knowledge base. Second, there is currently no standardized format for storing human GPS tracking data in a long-term archive or for sharing such data. This limitation makes collaboration and the development of open and reproducible methods in human mobility research challenging. There is a clear need to generate core open data standards and common vocabulary for human GPS tracking data that are programming language–agnostic, yet compatible with widely used tools in open data science languages (e.g., Python, R), to promote reproducibility and replicability in human mobility studies. Additionally, adhering to FAIR data principles, making data Findable, Accessible, Interoperable, and Reusable, ensures that these datasets are standardized and optimized for sharing and reuse.

**Fig. 1:**
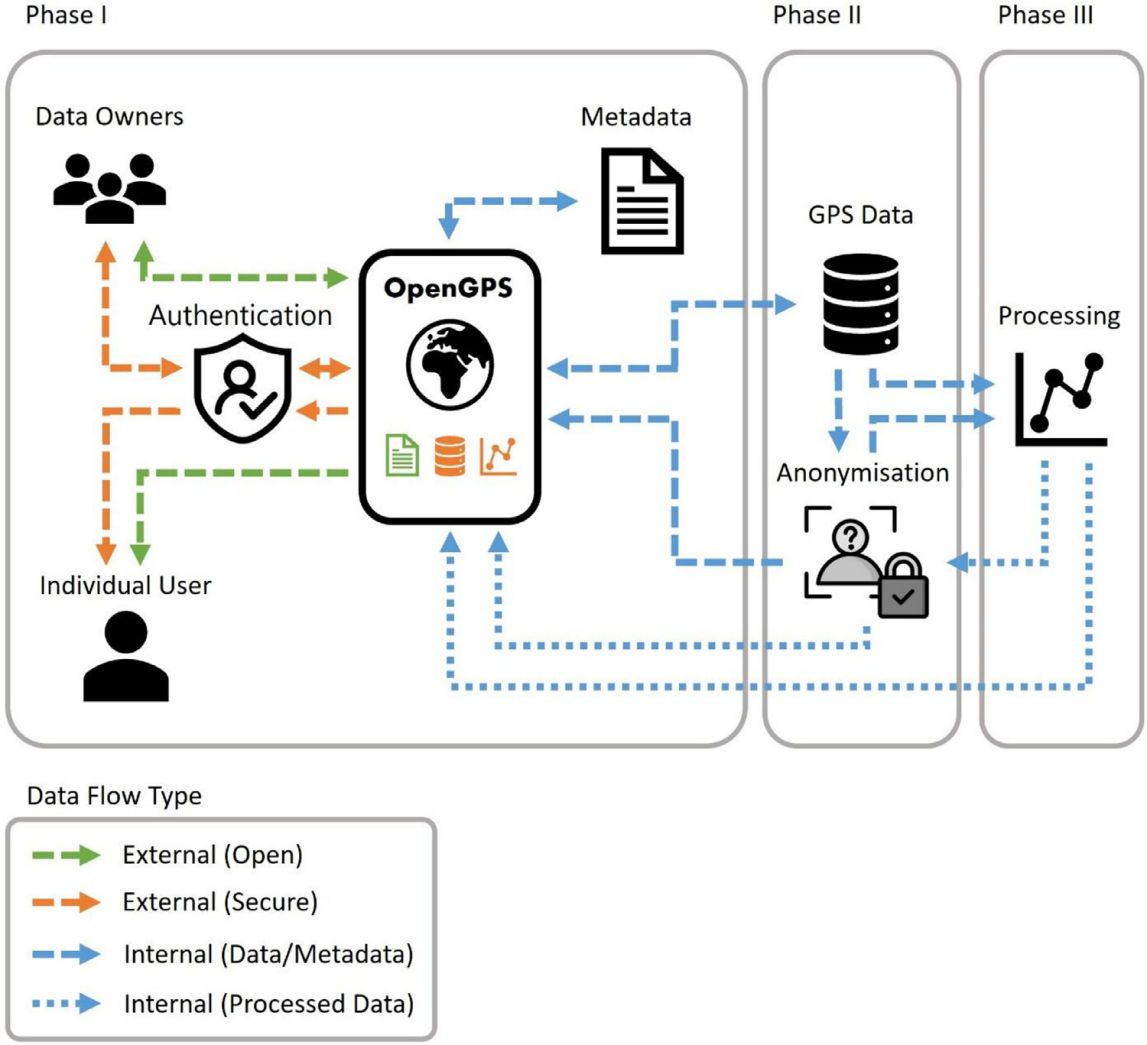
The OpenGPS project dashboard and workflow. Data owners will be able to securely upload project metadata and GPS tracking data. Project metadata will be openly available. Project GPS data will be secured using privacy preserving systems to protect sensitive location information. Multi-level sharing can be facilitated between teams and users.

To this end, we envision the development of OpenGPS as having three-phases, with each phase requiring increased engagement with the global community of scientists collecting, managing, processing, and analyzing GPS tracking data in human mobility studies (Fig. 1). Phase I of the OpenGPS will focus on the collection of metadata associated with GPS tracking studies globally. Phase I is currently ongoing. Phase II of the OpenGPS development will focus on archiving (storage) and sharing GPS tracking data between researchers. As such, the development of Phase II will require substantial buy-in from the global user community using GPS tracking for human mobility studies. To encourage contributions, we propose formal citation protocols to credit dataset authors, and co-authorship opportunities for dataset contributions. This idea is based on the sharing culture in movement ecology, which gives credit to data owners through automatical co-authorship on any papers that use their data. All contributions submitted during Phase I and Phase II will be reviewed by members of the OpenGPS core development team, with support from a network of volunteer researchers. Finally, our vision for Phase III is the incorporation of key analytical and exploratory tools and workflows directly into the OpenGPS system to facilitate collaborative efforts analyzing multiple datasets more quickly and efficiently. Therefore, Phase III aims to facilitate novel lines of questioning by combining datasets from multiple studies across geographical regions and over time.

Developing a sharing platform of this scope is a sizeable endeavour, requiring a buy-in and support from many different stakeholders. In this paper, we present the prototype of Phase I, developed as a pilot project, and outline the envisioned progression of Phases II and III, along with the key challenges that must be addressed for further development. We encourage the human mobility research community to engage with and contribute to this initiative, fostering collaboration and shaping the future of open and reproducible GPS tracking research.

### 2.1 Phase I: metadata

**Fig. 2:**
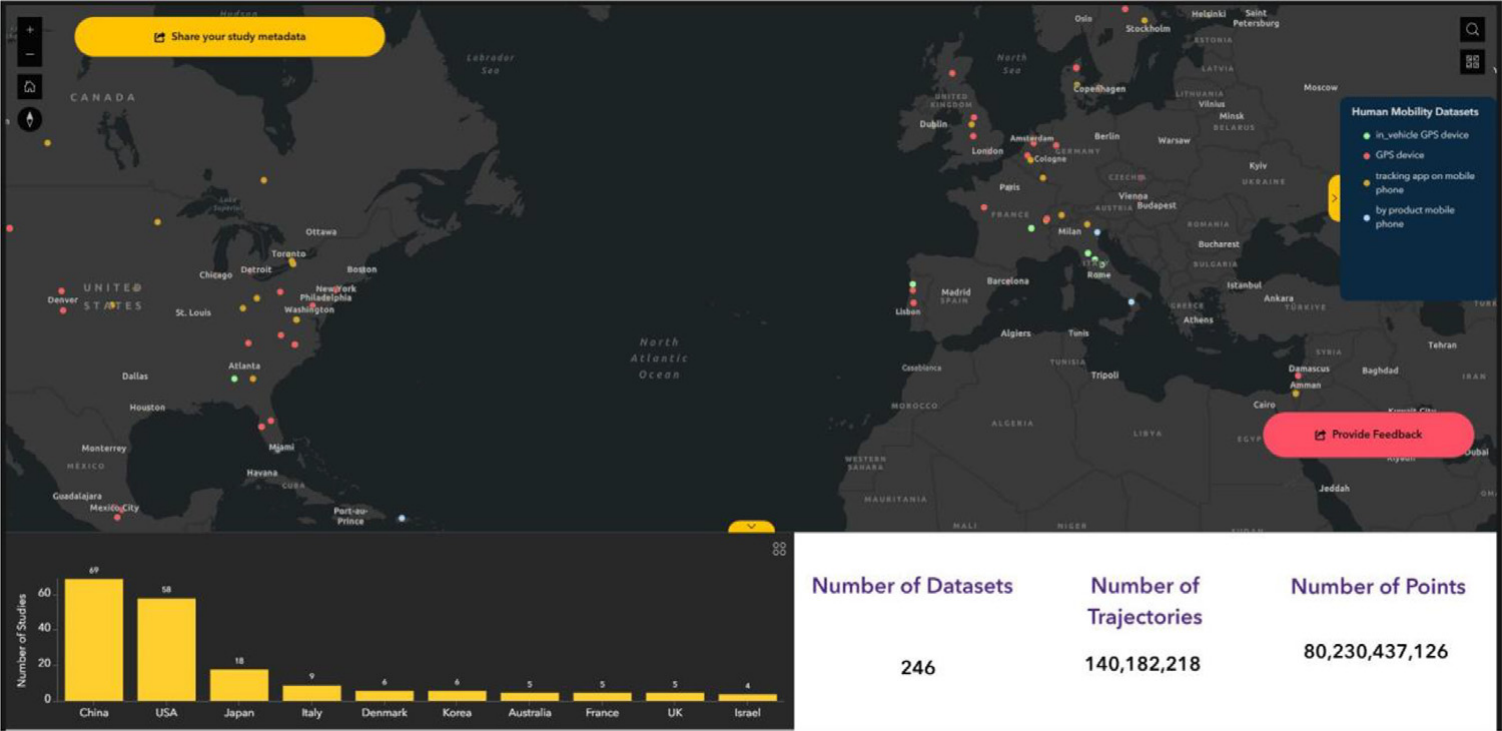
OpenGPS prototype dashboard (Link). Studies are arranged by location on the map and key study metadata can be queried.

Due to the sensitive nature of GPS tracking data, Phase I of the OpenGPS focuses exclusively on the collection of metadata from GPS tracking studies. To populate the initial prototype of OpenGPS, we conducted a broad search for existing GPS tracking studies focused on human mobility. This effort resulted in the collection of metadata from 241 studies worldwide (Fig. 2). Compiling this metadata enabled us to map current research practices, identify frequently used variables, and highlight areas of potential overlap, laying the groundwork for future efforts in data standardization and harmonization. For each study, we collected essential information including the primary investigator's name, contact information, and the study area. Information such as the start and end dates of data collection, the types of devices used, data sources, and the volume of data was stored where available. We also documented whether common attributes, typically derived from participant questionnaires, were included in the datasets. These attributes were selected based on the most shared information by researchers in their publications. To support future expansion and refinement of these attributes, the OpenGPS prototype includes a feedback form where users are asked to suggest the most relevant metadata fields they consider when evaluating the usefulness of a dataset. The collected data/attributes help determine the dataset’s relevance and usability for repository users. From this initial search, we begin to see the potential for combining studies across regions, city types, and study designs to answer increasingly sophisticated and novel questions about human mobility patterns.

Phase I helps developing a standardized framework for human mobility data. Drawing inspiration from bio-logging initiatives [26], we propose creating a common set of definitions and formats for key variables in GPS tracking studies. Such a standard would minimize inconsistencies in data recording (e.g., variations in units, naming conventions, or data types) and enable automated integration and analysis across studies. Moreover, by defining consistent transfer and aggregation protocols, we can streamline the movement of data between the OpenGPS and analytical tools, ensuring that complex datasets remain interoperable and their derived insights robust. This standardized approach not only enhances data clarity and usability but also lays the groundwork for scalable, reproducible research in human mobility studies.

To allow researchers to add to the OpenGPS database, the OpenGPS prototype has a simple form that allows project members to input detailed information about their study to further populate the database beyond those studies we initially identified. The studies are then screened by a member of the OpenGPS development team before becoming live points in the database. The OpenGPS prototype then facilitates the searching of studies based on characteristics and/or study regions and – importantly – provides contact information of the data holder to help facilitate new collaborations.

### 2.2 Phase II: archiving GPS data

The archiving of GPS tracking data will require the development of three key OpenGPS components. First, we need to develop a standardized data format that can be universally integrated into existing open data science workflows for storing and sharing GPS tracking data. Here we can draw on existing work on mobile databases and data formats that facilitate fast and efficient queries of mobility datasets (e.g., [27,28]). This component of OpenGPS will require flexibility in accommodating datasets with various spatial-temporal granularities and the inclusion of contextual covariates associated directly with the GPS tracking data [2].

The second component revolves around safeguarding individual location privacy; it is imperative that we incorporate methods for preserving individual location privacy where data is stored. A possible approach could involve leveraging the suite of methods that are currently available for masking or obfuscating individual tracking data (e.g., k-anonymity for location clustering, or differential privacy with noise injection) [29,30], AI-based privacy methods (e.g. federated learning, synthetic data generation) [31,32], or cryptographic algorithms (e.g., homomorphic encryption, zero-knowledge proofs) [33,34]. These methods each have their strengths, but also limitations when applied in isolation. Given that no single technique can fully address all privacy risks, especially against evolving re-identification attacks [[35], [36], [37]], we believe that a hybrid approach combining complementary methods offers the most promising path forward. OpenGPS will therefore allocate substantial effort toward the development, and implementation of cutting-edge, context-specific privacy solutions, adapted to legal and technological constraints in different regions. These solutions may involve using decentralized data methods to allow for data to be stored securely within individual countries and institutions to accommodate national data privacy regulations [38]. How such a system could be implemented remains an open area of research on both the regulatory and technical side of the problem. Compliance with regulations like the EU’s GDPR may require clarifying data controller responsibilities, obtaining valid consent for cross-border transfers, and potentially implementing a decentralized storage model for sensitive raw data.

The third and final component of Phase II of OpenGPS will involve methods for data sharing. Data sharing may be possible without the development of a robust data format or privacy preservation techniques simply by contacting data holders. However, implementing efficient methods for sharing GPS tracking data, including multi-level access for different user groups, will significantly enhance collaboration opportunities, facilitate novel research inquiries, and advance methodological development. This approach will enable selective data sharing and reduce the need for redundant data processing, thereby optimizing the data dissemination process.

In Phase II, our goal is to integrate the archiving of GPS tracking data into the OpenGPS platform as a standard component of research design and ethical review processes for human mobility studies worldwide. Specifically, we envision that researchers will outline their plans to utilize the OpenGPS platform during the research design phase, such as when applying for funding or undergoing ethical review. By incorporating this step early in the research process, participants can be fully informed about the final destination of their data and the geoprivacy protections in place. This proactive approach ensures that all stakeholders are aware of the data archiving procedures and privacy safeguards, promoting transparency and ethical compliance throughout the research lifecycle.

### 2.3 Phase III: analysis and workflows

A long-term goal is to integrate key analysis, processing steps and workflows into the OpenGPS system. This is not solely intended to simplify data handling but to support scenarios where legal or institutional constraints prevent raw data from being openly shared or downloaded. In such cases, OpenGPS would offer a secure, privacy-preserving environment where researchers can process data within the platform itself, mitigating the risks associated with data transfer. While the available toolset may be more limited than what is available within well-resourced laboratories, the standardized analytical functions and shared workflows are expected to significantly enhance accessibility, interoperability, and reproducibility, particularly for researchers operating in less resource-intensive environments.

We envision a system where GPS tracking data is automatically processed into stops, representing activities, and trips using a standardized method. Further, we hope to leverage widely available global datasets, such as OpenStreetMap (https://openstreetmap.org) to provide further context for GPS tracking data, such as associating stops and trips with points-of-interest [5], road types [39], and other features. The integration of such global external datasets has potential to standardize practices in context-aware movement analysis (CAMA) [40]. CAMA includes methodological integration of GPS tracking data and contextual information describing the environment or physiological state of the individual to uncover the reason behind specific movement patterns [41]. Moreover, there is substantial opportunity to incorporate exploratory spatial data analysis (ESDA) techniques to identify patterns, anomalies, and relationships within the data, further enriching the analytical process [42]. Automating the labeling of trips by mode of transit, which is often a crucial step in processing raw GPS data, is another significant opportunity [43]. Automating more sophisticated analysis may also be possible. For example, it is relatively straightforward to calculate different movement indicators (e.g., total travel time, total travel distance, and activity space) from raw and/or processed GPS tracking data. Such readily available indicators capture various dimensions of mobility that are often used in subsequent analyses [44]. Finally, the system could include advanced visualization tools to help researchers and stakeholders better understand and communicate mobility patterns and insights derived from the data. In parallel, we envision to provide APIs and libraries compatible with R (e.g., sf), Python (e.g., geopandas), and QGIS plugins, enabling seamless integration of OpenGPS data into existing geospatial workflows.

## 3 Research Potential of a Global Platform of GPS Tracking Studies

Establishing a comprehensive global geospatial archiving and processing platform for GPS tracking studies offers several benefits for the scientific community. First by facilitating enhanced collaboration among researchers in this field, OpenGPS will serve as a platform for direct communication and knowledge sharing by providing contact information for data holders associated with each dataset. This promotes the initiation of potential collaborations, allowing researchers to exchange ideas and work together. Such collaborations can lead to advancements in understanding human mobility dynamics by leveraging multiple researchers' collective expertise and resources. Additionally, this platform can maximize the utility of existing datasets by enabling broader access and reuse, thereby reducing the costs and redundancy associated with new data collection efforts.

Currently, research collaborations heavily depend on the researchers' networks, which are often limited to countries with greater resources and funding. The OpenGPS platform has potential to play a crucial role in help dismantling these barriers, enabling scientists from other global regions to establish stronger connections and collaborations. By providing an equitable platform for communication, and subsequently sharing data, the OpenGPS should help researchers from diverse geographical and economic backgrounds with the opportunity to contribute to and benefit from global research efforts, promoting a more inclusive scientific community. Additionally, we aim to provide server-side data processing options that minimize local computing needs, helping researchers in lower-income regions with limited resources, and ensuring that the platform remains accessible to diverse users.

Another benefit of OpenGPS is its ability to provide a global perspective on the state of human mobility research using GPS tracking data. Our initial search indicates that the majority of GPS tracking studies to date are predominantly conducted in the Global North. The global perspective of OpenGPS may help identify research gaps and areas that have received less attention, prompting researchers to explore and investigate human mobility patterns in previously understudied regions, with different study designs, and/or target new or different questions. Additionally, the repository’s global coverage highlights the potential for regional comparisons and the examination of factors that may influence human mobility. Such analyses could contribute to addressing biases in human mobility research.

The development of OpenGPS presents a unique opportunity to move toward a dynamic, continuously evolving record of human movement. By systematically archiving GPS tracking studies, integrating metadata, and enabling standardized data formats, OpenGPS lays the groundwork for capturing large-scale patterns of human mobility over time. Unlike static datasets that provide snapshots of movement at specific points, a continuously updated repository of GPS tracking studies can facilitate long-term analyses of mobility shifts due to urban development, socio-economic changes, and global events. This dynamic system would enable researchers to explore human movement at different scales, from local travel behaviours to large-scale migration trends, while preserving contextual details.

We strongly believe that an initiative like OpenGPS will encourage researchers to adopt common designing studies, and storing, sharing, and processing GPS data. Such a level of consistency in data storing, formatting, and processing is currently not occurring and is crucial for comparing across studies. By collecting and presenting a set of detailed metadata attributes OpenGPS will further embed the importance of comprehensive metadata alongside GPS tracking datasets.

## 4 Governance, Challenges, and the Path Forward

Realizing the full potential of the OpenGPS platform, spanning standardized data collection and metadata sharing, integrated analytical workflows, and sustainable archiving, demands a coordinated governance framework. A dedicated body, analogous to initiatives in other scientific domains such as the International Bio-Logging Society, must be established to represent the human mobility research community and drive the implementation of community standards. This organization would serve as a central authority, uniting diverse stakeholders, researchers, data owners, funding agencies, permitting authorities, publishers, and governmental data centres, to secure a formal mandate and sustainable funding streams. By actively shaping data-sharing policies and incentivizing open practices, such a governing body would not only streamline the integration and reproducibility of human mobility data but also ensure the long-term viability and impact of OpenGPS on research and policy development [45]. While OpenGPS cannot resolve all legal and institutional barriers to data sharing, it provides a critical foundation for coordinated action and a trusted network to build upon.

A major obstacle to advancing OpenGPS is that, despite incentives from journals and funding agencies [46], a considerable portion of human mobility GPS datasets remains unarchived— in our search of 241 studies, less than 3% had a publicly accessible URL—significantly restricting their accessibility and long-term utility. On the one hand, many researchers feel that their project objectives are met without the need for public data sharing, and privacy concerns often deter them from releasing sensitive location data. On the other hand, institutional and regulatory frameworks may unintentionally discourage data sharing, either by not requiring public archiving as a condition of study approval or by imposing restrictive data management practices to avoid potential conflicts. This dual challenge leads to fragmented datasets, undermining the reproducibility and broader utility of human mobility research.

A viable path forward involves a coordinated push from governing bodies and institutional stakeholders. Integrating mandatory data registration into existing ethical review and funding processes can serve as a powerful incentive for researchers, ensuring that data archiving becomes an integral part of study approval [47]. By aligning these requirements with current permitting practices, research institutions, governmental agencies, funders, and publishers can promote the adoption of standardized, privacy-preserving data repositories, such as our proposed platform, OpenGPS. Furthermore, demonstrating tangible benefits as pull factors, including automated data integration, standardized formats, and advanced analytical tools, will help build trust and encourage widespread participation.

While a fully centralized repository is our ideal vision for OpenGPS, we recognize that many institutions and countries impose rules that limit external data storage and sharing. In light of these constraints, an advanced iteration of our architecture could adopt a federated architecture [48,49], allowing for local hosting of sensitive data while still connecting to the broader OpenGPS network. In this approach, individual institutions would maintain control over their raw data on local servers that comply with their specific regulatory and security requirements. At the same time, standardized metadata and non-sensitive information would be shared with the central system, ensuring that researchers can collaborate and integrate data without compromising local governance policies.

This connected, distributed framework represents an evolution of our current architecture, combining the strengths of centralized coordination with the flexibility of local data management. Although implementing such a hybrid system poses significant technical and administrative challenges, and may not be immediately feasible for all institutions, it offers a promising pathway forward. By ensuring that essential metadata remains accessible through standardized interfaces and that sensitive data is securely hosted locally, OpenGPS can foster collaboration and reproducible research while respecting the diverse legal and institutional landscapes that govern human mobility data.

Finally, a development of a global platform at this scale requires input from the entire research community. We therefore aim to get feedback from the GPS tracking community to gain a better understanding of how Phase II (and potentially Phase III) of OpenGPS could be further enhanced to serve the community and foster collaboration and novel lines of study. To facilitate this, we have created a feedback form that will be continuously updated throughout each phase of the project which can be accessed on the platform. Further, our vision and a request for the feedback will be promoted widely in the related communities, for example through workshops at relevant conferernces (GIScience, SIGSPATIAL, AGILE and others) and through direct contact with researchers whom we identified in the preparation of Phase I. To further strengthen community participation, we also plan to organize focused workshops on topics such as GPS tracking data standardization, privacy-preserving sharing practices, and data interoperability. In parallel, we envision proposing special issues in relevant journals, such as Data in Brief, to encourage the publication of open human mobility datasets and promote best practices for documentation and sharing. These coordinated efforts will help foster a collaborative and standards-driven community around human mobility research, ensuring that the development of OpenGPS is informed by the needs and contributions of its users.

## 5 Conclusion

The development of OpenGPS is currently in its preliminary phases, focusing on the Phase I component of GPS tracking study metadata. We see this first step as crucial, because in and of itself, an archive of GPS tracking study metadata will be highly beneficial to the field by providing information on study locations, data characteristics, study design, and data holders. This rich metadata repository will enable researchers to quickly identify relevant datasets, understand the scope and context of previous studies, and facilitate new collaborations. By creating a centralized, accessible repository, we are setting the stage for a transformative leap in research capabilities, ensuring that subsequent phases of OpenGPS are built on a robust and well-organized knowledge base. This foundational step is indispensable for fostering an environment of open data sharing, reproducibility, and innovative research methodologies in the study of human mobility. We acknowledge, however, that extending OpenGPS to include privacy-preserving data storage and integrated analytics requires substantial technical, institutional, and financial commitments. Each component—metadata curation, privacy-focused infrastructure, and built-in analytics—demands ongoing research and collaboration with the broader community. This paper is therefore a call to the human mobility research community to adopt the open sharing culture and contribute to the development of the OpenGPS platform.

## Data and Codes Availability Statement

The list of all 241 studies reviewed in the comprehensive review can be accessed through the following link: https://figshare.com/s/d7234bbe52a380ef9385. The OpenGPS prototype was developed using Esri’s ArcGIS Experience Builder. No custom code was required for this stage of development.

## Ethics Statement

The authors confirm that the current work does not involve human subjects, animal experiments, or any data collected from social media platforms. The authors have read and followed the ethical requirements for publication in Data in Brief.

## CRediT author statement

**Milad Malekzadeh:** Conceptualization, Methodology, Software, Validation, Formal Analysis, Investigation, Data Curation, Writing – Original Draft, Writing – Review & Editing, Visualization, Project Administration, **Hui Jeong Ha:** Conceptualization, Methodology, Validation, Investigation, Data Curation, Writing – Review & Editing, **Katarzyna Sila-Nowicka:** Conceptualization, Writing – Review & Editing, Visualization, **Vanessa Brum-Bastos:** Conceptualization, Writing – Review & Editing, **Jinhyung Lee:** Conceptualization, Writing – Review & Editing, **Urška Demšar:** Conceptualization, Writing – Review & Editing, Supervision, Project Administration, **Jed A. Long:** Conceptualization, Methodology, Resources, Writing – Original Draft, Writing – Review & Editing, Supervision, Project Administration.

## Funding statement

This research did not receive any specific grant from funding agencies in the public, commercial, or not-for-profit sectors.

## Declaration of Competing Interest

The authors declare that they have no known competing financial interests or personal relationships that could have appeared to influence the work reported in this paper.
